# Free water corrected diffusion tensor imaging discriminates between good and poor outcomes of comatose patients after cardiac arrest

**DOI:** 10.1007/s00330-022-09245-w

**Published:** 2022-11-24

**Authors:** Hanneke M. Keijzer, Marco Duering, Ofer Pasternak, Frederick J. A. Meijer, Marlous M. L. H. Verhulst, Bart A. R. Tonino, Michiel J. Blans, Cornelia W. E. Hoedemaekers, Catharina J. M. Klijn, Jeannette Hofmeijer

**Affiliations:** 1 Department of Neurology, Rijnstate Hospital, P.O. box 9555, 6800 TA Arnhem, The Netherlands; 2 Department of Neurology, Donders Institute for Brain, Cognition, and Behaviour, Radboud University Medical Centre, 6500 HC Nijmegen, The Netherlands; 3 Institute for Stroke and Dementia Research (ISD), University Hospital LMU, 81377 Munich, Munich Germany; 4 Medical Image Analysis Centre (MIAC AG), Basel and qbig, Department of Biomedical Engineering, University of Basel, CH-4051 Basel, Switzerland; 5 Departments of Psychiatry and Radiology, Brigham and Women’s Hospital, Harvard Medical School, Boston, MA 02115 USA; 6 Department of Radiology and Nuclear Medicine, Radboud University Medical Centre, 6500 HC Nijmegen, The Netherlands; 7 Department of Clinical Neurophysiology, Faculty of Science and Technology, University of Twente, 7522 NB Enschede, The Netherlands; 8 Department of Radiology, Rijnstate Hospital, 6800 TA Arnhem, The Netherlands; 9 Department of Intensive Care Medicine, 6800 TA, Rijnstate Hospital, Arnhem, The Netherlands; 10 Department of Intensive Care Medicine, Radboud University Medical Centre, 6500 HC Nijmegen, The Netherlands

**Keywords:** Brain edema, Brain ischaemia, Brain imaging, Cardiac arrest, MRI

## Abstract

**Objectives:**

Approximately 50% of comatose patients after cardiac arrest never regain consciousness. Cerebral ischaemia may lead to cytotoxic and/or vasogenic oedema, which can be detected by diffusion tensor imaging (DTI). Here, we evaluate the potential value of free water corrected mean diffusivity (MD) and fractional anisotropy (FA) based on DTI, for the prediction of neurological recovery of comatose patients after cardiac arrest.

**Methods:**

A total of 50 patients after cardiac arrest were included in this prospective cohort study in two Dutch hospitals. DTI was obtained 2–4 days after cardiac arrest. Outcome was assessed at 6 months, dichotomised as poor (cerebral performance category 3–5; *n* = 20) or good (*n* = 30) neurological outcome. We calculated the whole brain mean MD and FA and compared between patients with good and poor outcomes. In addition, we compared a preliminary prediction model based on clinical parameters with or without the addition of MD and FA.

**Results:**

We found significant differences between patients with good and poor outcome of mean MD (good: 726 [702–740] × 10^-6^ mm^2^/s vs. poor: 663 [575–736] × 10^-6^ mm^2^/s; *p* = 0.01) and mean FA (0.30 ± 0.03 vs. 0.28 ± 0.03; *p* = 0.03). An exploratory prediction model combining clinical parameters, MD and FA increased the sensitivity for reliable prediction of poor outcome from 60 to 85%, compared to the model containing clinical parameters only, but confidence intervals are overlapping.

**Conclusions:**

Free water-corrected MD and FA discriminate between patients with good and poor outcomes after cardiac arrest and hold the potential to add to multimodal outcome prediction.

**Key Points:**

*• Whole brain mean MD and FA differ between patients with good and poor outcome after cardiac arrest.*

*• Free water-corrected MD can better discriminate between patients with good and poor outcome than uncorrected MD.*

*• A combination of free water-corrected MD (sensitive to grey matter abnormalities) and FA (sensitive to white matter abnormalities) holds potential to add to the prediction of outcome.*

**Supplementary Information:**

The online version contains supplementary material available at 10.1007/s00330-022-09245-w.

## Introduction

Postanoxic encephalopathy is the main cause of death in comatose patients after cardiac arrest and successful resuscitation. Approximately 50% of patients admitted to the intensive care unit (ICU) will never regain consciousness because of severe postanoxic encephalopathy [1, 2]. Early prediction of neurological outcome is important for multiple reasons. Accurate early prediction of poor outcome prevents patients from undergoing futile long-term and expensive treatment in an ICU, whereas premature treatment withdrawal is avoided for patients with relevant chances of recovery. Over the past years, substantial research efforts have resulted in prognostic electrophysiological [3, 4] and imaging parameters [5, 6]. However, current clinical prognostic measures yield only moderate predictive properties, with a reported accuracy of prediction of good or poor outcome in 30–70% of patients [3, 5–8].

The pathophysiological processes of postanoxic encephalopathy are important to understand when searching for possible predictors, but complex [9, 10]. Cerebral ischaemia starts with the failure of synaptic neurotransmission [11]. In case of severe or lasting ischaemia and ATP depletion, cell swelling or cytotoxic oedema occurs within minutes to days after cardiac arrest. Starting after one to two days, dysfunction of the blood-brain barrier may result in an inflow of plasma in the interstitial space, called vasogenic oedema [10].

Diffusion-weighted imaging (DWI) is sensitive to diffusion restriction within the brain [12]. Diffusion Tensor Imaging (DTI) is a model that combines several DWI measurements collected along different orientations. DTI can characterise both the magnitude and the direction of water diffusion, thus probing the brain tissue microstructure [13]. Two DTI measures are of particular interest: mean diffusivity (MD) reflects the magnitude of diffusion and fractional anisotropy (FA) its directionality. Cytotoxic oedema leads to diffusion restriction, which is reflected by lower MD values, most prominent at 2–5 days after cardiac arrest [12, 14, 15]. Therefore, optimal discrimination by diffusion imaging is expected in this time window.

On the other hand, vasogenic oedema may increase MD due to the influx of water in the interstitial space [16]. Also, the dilution effects of vasogenic and cytotoxic oedema within the white matter results in a decrease in FA [17].

CSF has vastly different diffusion properties than brain tissue and is thus a prominent confounder of diffusion metrics in regions susceptible to partial volume effects. DTI analyses corrected for free water might provide a more specific measure of tissue damage by minimising the effect of CSF and vasogenic oedema [18, 19]. This is especially relevant in cortical areas, that are sensitive to hypoxia, but suffer greatly from CSF partial volume effects.

Previous studies have shown group differences and promising predictive values of diffusivity and FA between patients with good and poor outcomes. However, the overall quality of evidence is limited due to the often-retrospective study designs, variable timing of MRI, and a broad range of reported predictive values [5, 6, 8, 20–24].

Here, we study free water-corrected MD and FA in patients with good and poor outcomes after cardiac arrest and evaluate the potential to add to the multimodal prediction of neurological outcome. To estimate the additional value of MD and FA as predictors, we combine these with several parameters that are used in current clinical care in an exploratory prediction model for neurological outcome after cardiac arrest.

## Materials and methods

We performed a prospective multicentre cohort study on the outcome prediction of comatose patients after cardiac arrest. Patients were included in two Dutch hospitals, Rijnstate hospital and Radboud University Medical Centre (Radboudumc). The study was approved by the Committee on Research Involving Human Subjects region Arnhem-Nijmegen and registered on clinicaltrials.gov (identifier: NCT03308305). For the current analyses, we used brain MRI data of day 3 ± 1 after cardiac arrest, collected between June 2018 and November 2020.

### Patients

We included consecutive patients after permission from their legal representatives within 72 h after cardiac arrest. Inclusion criteria were as follows: cardiac arrest based on a cardiac cause (including pulmonary embolism), Glasgow Coma Scale ≤ 8 at admission, age ≥ 18 years, and admission to the ICU. Exclusion criteria were: pregnancy, life expectancy < 24 h post cardiac arrest, any known progressive brain illness, pre-existing dependency in daily living, or a contraindication to undergo MRI scanning (e.g. presence of a pacemaker or foreign metal objects).

Patients were treated according to international guidelines [8, 25], as described in the local ICU protocols. Targeted temperature management at 36°C (Rijnstate Hospital) or 32–34°C (Radboudumc) was induced after arrival at the ICU and maintained for 24 h. Upon rewarming, normothermia was actively maintained. Patients generally received (a combination of) propofol, midazolam, morphine, or sufentanil for sedation and analgesia.

Withdrawal of life-sustaining treatment (WLST) was considered at ≥ 72 h after cardiac arrest, during normothermia, and off sedation. Decisions on WLST were based on international guidelines including the absence of brainstem reflexes, treatment-resistant myoclonus, and bilateral absence of somatosensory evoked potentials (SSEPs) after reaching normothermia and depletion of sedative medicines [25]. Since April 2019, visual classification of the EEG is part of the Dutch guideline “prognosis of postanoxic coma.” Poor outcome is predicted with suppressed EEG at > 12 h after cardiac arrest or low voltage, burst suppression with identical bursts, or generalised periodic discharges on a suppressed background at > 24 h [26]. Decision-making regarding WLST always took into account the specific background of the patient and involved consultation of a multidisciplinary team and the patient’s family. In case of doubt, the decision was postponed and the patient was re-evaluated at a later time. MRI was never included in decisions on treatment withdrawal.

The primary outcome measure was “good” (CPC 1–2, no to mild neurological impairments) or “poor” (CPC 3–5, severe neurological impairments, vegetative state or death) neurological outcome at six months after cardiac arrest, as recommended by the International Liaison Committee on Resuscitation [27]. CPC scores were estimated by a standardised telephone interview based on the EuroQol-6D questionnaire, by an investigator (H.K., M.V.) blinded to the clinical course of the patient, EEG, and MRI readings.

### Data acquisition

All patients underwent a 3-T MRI scan at 3 ± 1 days after cardiac arrest (Philips Ingenia (Rijnstate) or Siemens Skyra (Radboudumc), one scanner per site). For this study, we used 3D T1-weighted (Siemens: MPRAGE, voxel size 0.9 × 0.9 × 1.0 mm, Philips: 3D TFE, voxel size 1.0 × 1.0 × 1.0 mm), and 2D diffusion-weighted imaging sequences (Siemens: voxel size 2.0 × 2.0 × 2.0 mm, 1 non-diffusion weighted image, 30 images with a b-value of 1000 s/mm^2^, TR/TE 9700/95 ms; Philips: voxel size 2.0 × 2.0 × 2.0 mm, 1 non-diffusion weighted image, 32 images with a b-value of 1000 s/mm^2^, TR/TE 9000/95 ms). Further details are listed in Supplementary Table 1.

### Data analyses

Pre-processing of the DTI data consisted of the reduction of thermal noise and removal of Gibbs artefacts (MRtrix version 3.0 [28], dwidenoise and dwidegibbs), followed by correction for Eddy current distortions and motion (“EDDY”; fMRI brain Software Library (FSL); v6.0.2 [29]). Raw data and results of pre-processing were visually checked for quality and erroneous results and artefacts.

Diffusion measures were estimated both with and without free water correction. Diffusion models were calculated based on a nonlinear regularised minimisation process implemented in MATLAB (version R2016a, The MathWorks Inc.) [18]. For conventional DTI, a single tensor was directly fit on pre-processed data. For free water correction, a model with two tensors, representing two different diffusion compartments, was fit in each voxel. A fixed, isotropic tensor was used to model freely diffusing extracellular water (free water compartment). A second tensor was used to model the remaining signal, i.e. the signal originating from restricted water in tissue. Free water-corrected FA and MD were calculated from the tissue compartment’s tensor.

Individual FA maps were registered to a standard space (FMRIB58_FA) using the Tract-Based Spatial Statistics pipeline in FSL [30]. MD maps were co-registered to standard space using the subject-specific transformations obtained with FA maps. Voxel by voxel group comparisons of MD and FA between patients with good and poor outcome were performed using the FSL function “randomize” including the Threshold-Free Cluster Enhancement option [31]. This resulted in a family-wise error-corrected group difference map, where clusters with a *p* value < 0.01 were considered significantly different between patients with good and poor outcome. Patients with pre-existing structural abnormalities, such as atrophy, were excluded from the voxel-by-voxel comparisons, because of limited precision for registration to the standard space.

In all patients, we created whole brain masks from the T1 image using FSL’s Brain Extraction Tool (BET) and linearly registered those to the diffusion images in native space. We calculated mean MD and FA within these masks, using in-house created MATLAB scripts and FSLmaths. For the MD images uncorrected for free water, all voxels with ADC < 200 × 10^-6^ or > 2000x10^-6^ mm^2^/s were removed to exclude artefacts and influence of cerebral spinal fluid (CSF) [5, 15, 32]. In addition, we calculated the percentage of voxels with MD < 450 × 10^-6^ mm^2^/s within the whole brain mask (%MD_450_). This cut-off value was established after visual inspection of the MD images and corresponds with previously proposed cut-off values of the ADC [33, 34].

EEGs were classified offline and blinded to the timing of the epoch, the patient’s clinical status, MRI, and outcome. A computer randomly presented 5-minute artefact-free EEG epochs at 6, 12, 24, 36, 48, and 72 h after cardiac arrest to two reviewers (H.K., M.V.). EEG registrations were classified into three categories: (I) suppressed pattern with or without superimposed synchronous activity ≥ 24 h, (II) continuous activity ≤ 12 h, or (III) other patterns (Supplementary Table 2) [3, 35]. In case of disagreement, a consensus was determined by consultation with a third reviewer (J.H.).

### Statistical analyses

Data are presented as mean and standard deviation (SD) when normally distributed, or median [interquartile range (IQR)], otherwise. For group comparisons, we used chi-squared, student t-tests, or Mann-Whitney U tests, where appropriate. *p* values < 0.05 were assumed statistically significant. Effect sizes were calculated using Cohen’s d. Results of the voxel-by-voxel comparisons are presented as spatial maps of areas with statistically significant differences between patients with good and poor outcome at a *p* value < 0.01.

To estimate the potential additional value of MD and FA for multimodal prediction of neurological outcome, we created four different logistic regression models using 10-fold cross-validation. The first model is the clinical model, consisting of three continuous (age, duration of the arrest, first measured lactate) and two categorical (sex, EEG classification) parameters, currently used in clinical assessment. The second model consisted of mean FA and MD after free water correction. The third model combined the clinical model with mean FA and mean MD. We then performed a sensitivity analysis with the fourth model, replacing mean MD with %MD_450_.

Predictive values of the models were evaluated using receiver operating characteristics (ROC), area under the curve (AUC), maximal sensitivity to detect poor outcome without false positives, and sensitivity to predict good outcome with 90% specificity, in line with the recommendations in the European guideline [8].

Statistical analyses were performed using R version 3.5.3 and MATLAB.

## Results

Of 261 screened comatose patients after cardiac arrest, 50 patients were included, and 20 (40%) had a poor outcome. The main reasons for exclusion were a non-cardiac cause (*n* = 44), and no permission from the legal representative (*n* = 48; Supplementary Figure 1).

Patient characteristics are listed in Table 1. Four subjects (two with poor outcome) were excluded from the voxel-based comparisons, because of structural deficits hampering registration to the standard space. These included large ventricles in three elderly patients and one patient with a recent stroke.

**Table 1:** Baseline characteristics

Characteristic	Good outcome (*n* = 30)	Poor outcome (*n* = 20)	*p* value
Age	56 ± 11	67 ± 11	< 0.01
Male	25 (83%)	14 (70%)	0.26
OHCA	30 (100%)	20 (100%)	NA
Shockable first rhythm	30 (100%)	15 (75%)	< 0.01
Duration of resuscitation (min)	12 [10–15]	23 [19–31]	< 0.01
First lactate measured	3.6 [2.7–6.7]	4.1 [3.1–5.4]	0.57
GCS Motor score ≤ 3 at day 3	1 (3%)	15 (75%)	< 0.01
Bilaterally absent pupillary light response ≥ 72 h	0 (0%)	4 (20%)	0.01
SSEP performed	0 (0%)	16 (80%)	< 0.01
Absent SSEP response	0 (0%)	4 (25%)	0.01
EEG: continuous ≤ 12 h	15 (50%)	1 (5%)	< 0.01
EEG: suppressed pattern with or without superimposed synchronous activity ≥ 24 h	0 (0%)	3 (15 %)	0.03
Time to MRI (h)	76 ± 24	77 ± 25	0.94
CPC score:			
- 1	13 (43)	0	
- 2	17 (57)	0	
- 3	0	3 (15)	
- 4	0	0 (0)	
- 5	0	17 (85)	

Outcome is based on 6-month follow-up. Data are presented as n (%) for dichotomous variables, mean ± sd for normally distributed continuous variables, and median [IQR] otherwise. *CPC*, cerebral performance category; *OHCA*, out-of-hospital cardiac arrest

### Mean diffusivity

**Fig. 1:**
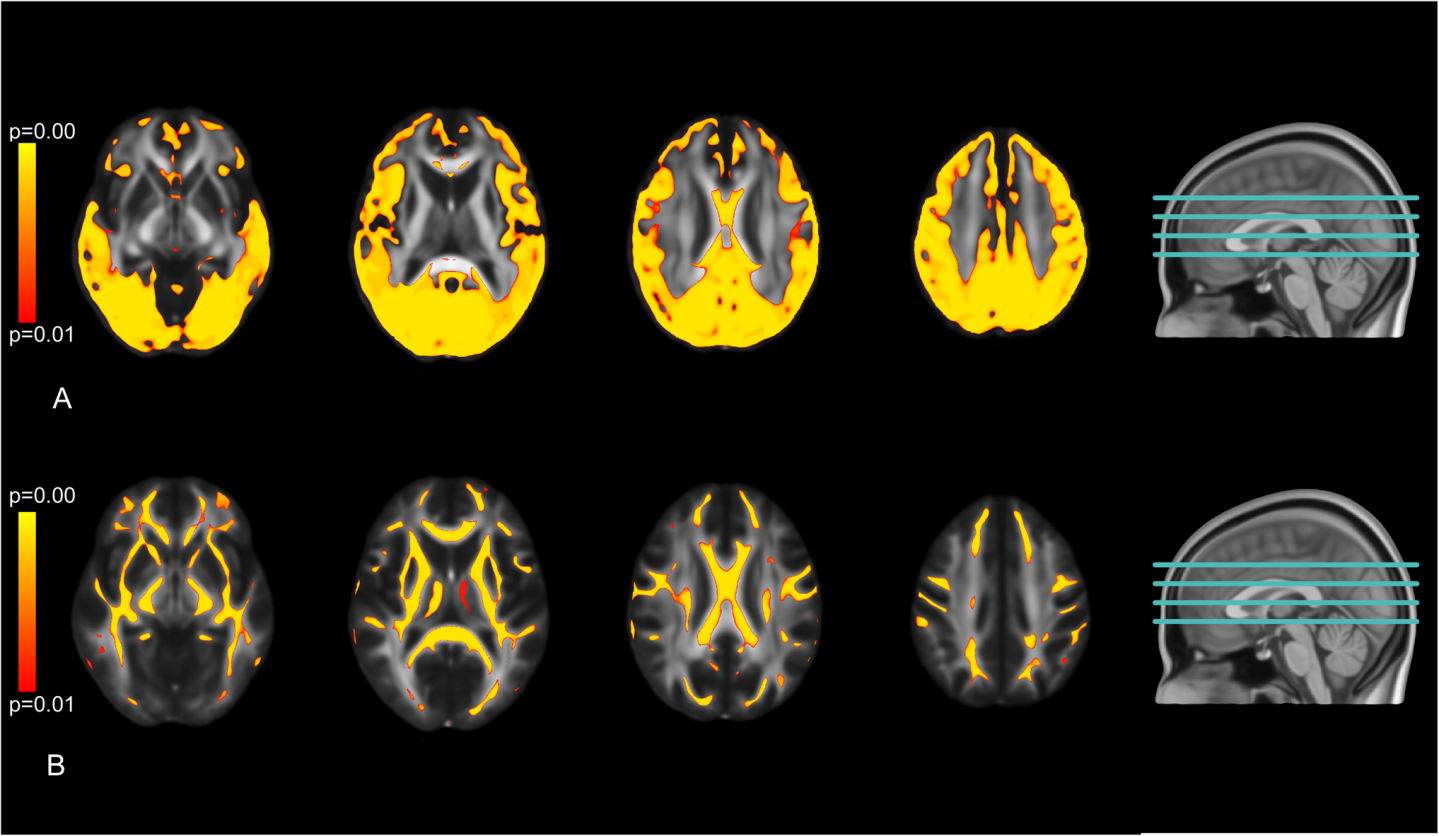
**A** spatial distribution of differences in mean diffusivity and (**B**) fractional anisotropy after free water correction. Coloured areas show brain areas where patients with poor neurological outcome show significantly lower values than patients with good neurological outcome (*p* < 0.01)

**Fig. 2:**
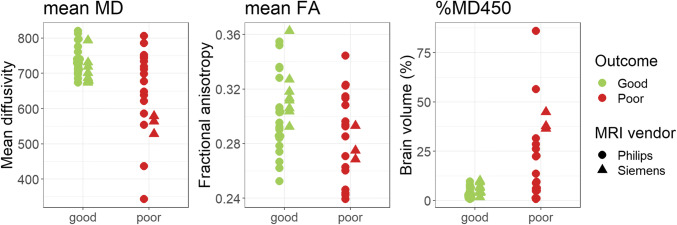
Whole-brain mean diffusivity (MD), fractional anisotropy (FA), and percentage of brain volume with MD < 450 × 10^-**6**^ mm^**2**^/s after free water correction for individual patients with good and poor outcome, differentiated by scanner site

Patients with poor outcome had lower MD in large parts of the cortical grey and white matter than patients with good outcome in voxel-based comparisons (Fig. 1A). These differences were most prominent in the occipital and parietal regions. The deep grey nuclei and deep white matter showed no differences in MD between patients with good and poor outcome. In patients with poor outcome, the median MD of the whole brain was lower than in patients with good outcome, with a large effect size (Table 2). MD values of Siemens and Philips MRI were comparable (Fig. 2A).

**Table 2:** Whole-brain mean diffusivity, fractional anisotropy, and percentage of brain volume with mean diffusivity below the threshold of 450*10^-**6**^ mm^**2**^/s

Parameter	Good outcome	Poor outcome	*p* value	Effect size
Mean diffusivity*	726 [702–740]	663 [575–736]	0.01	1.06 (0.44–1.67)
Fractional anisotropy	0.30 ± 0.03	0.28 ± 0.03	0.03	0.65 (0.06-1.24)
%MD_450_ (%)	3.4 [1.8–6.4]	18.0 [5.8–32.8]	< 0.01	1.31 (0.67–1.95)

Data are represented as *n* (%) for dichotomous variables, mean ± sd for normally distributed continuous variables and median [IQR] otherwise. Effect size is depicted as Cohen’s d (95% CI). *MD*, mean diffusivity; *%MD*_*450*_, brain volume with mean diffusivity below the threshold of 450 × 10^-6^ mm^2^/s; *MD × 10^-6^ mm^2^/s

Repeating analyses with MD uncorrected for free water yielded smaller brain areas with group differences in the voxel-wise analysis, and smaller effect sizes in the whole brain analysis (Cohen’s d 1.06 and 0.84 with and without free water correction respectively, Table 2; Supplementary Table 3). Therefore, only free water corrected measures were included in all the following analyses.

The %MD_450_ was higher in patients with poor neurological outcome than in those with good outcome (18% vs. 3%, Table 2). In 55% of the patients with poor outcome, > 11% of the brain showed an MD < 450 × 10^-6^ mm^2^/s, versus 0% in the patients with good outcome.

### Fractional anisotropy

Patients with poor outcome had widespread lower FA values in the cerebral white matter than patients with good outcome in voxel-based comparisons (Fig. 1B). We found no differences in FA values in the cortex and deep grey nuclei.

Furthermore, the mean FA of the whole brain was lower in patients with poor outcome than in patients with good outcome, with a medium effect size (Table 2). FA values of Siemens and Philips MRI were comparable (Fig. 2B).

### Potential predictive value

**Fig. 3:**
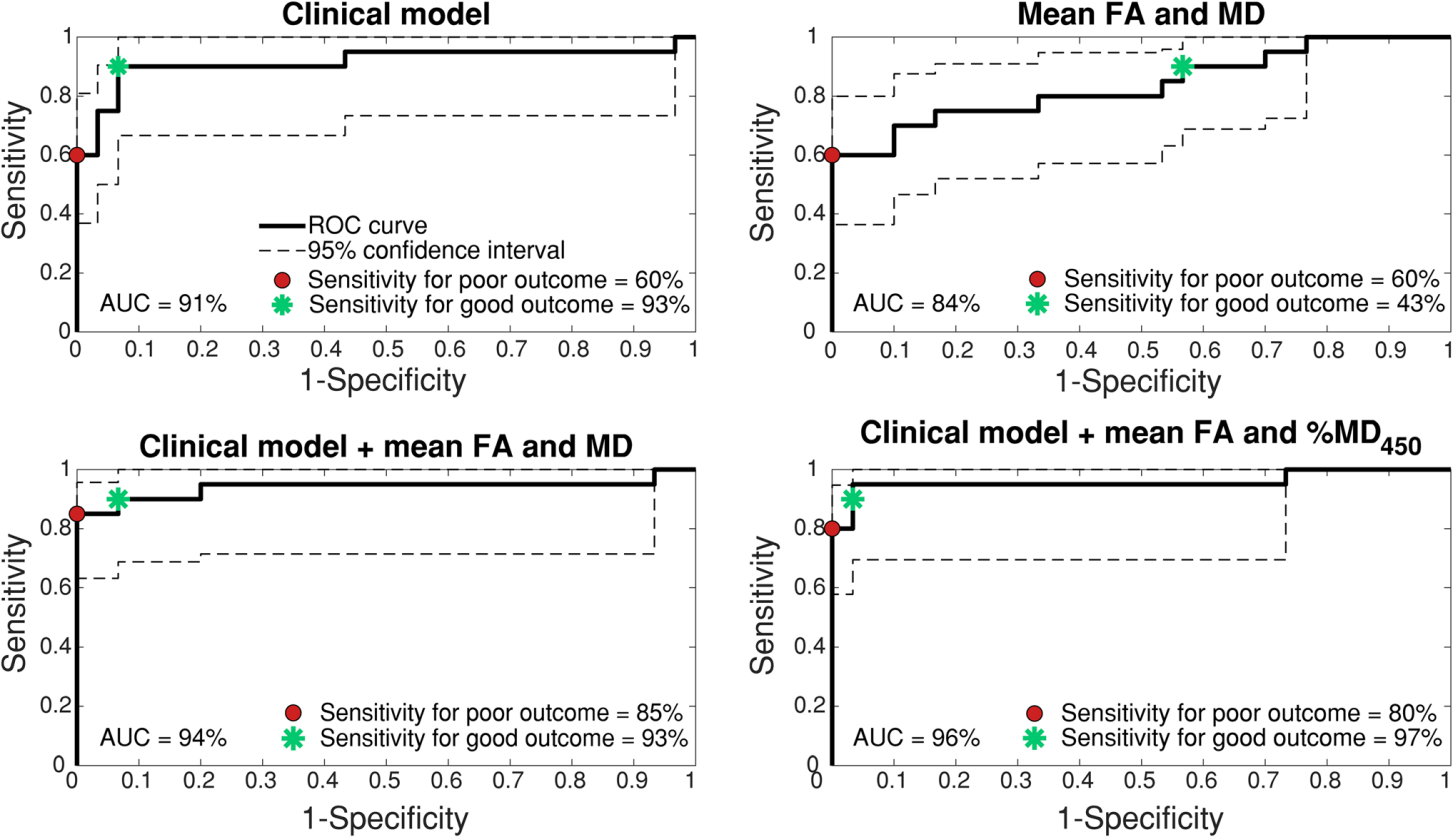
Receiver Operated Characteristic curves with 95% confidence interval of 4 logistic regression models with 10-fold cross-validation. The clinical models are based on age, sex, duration of resuscitation, shockable first rhythm, first measured lactate, and EEG classification. Specificity level for poor outcome is set at 100%, for good outcome at 90%. %MD_450_, percentage of brain volume with MD < 450 × 10^-6^ mm^2^/s; AUC, area under the curve

The clinical model was able to predict good outcome with 93% (78–100 95% CI) sensitivity at 90% specificity and poor outcome with 60% (37–80%) sensitivity at 100% specificity. Prediction model two, consisting of DTI measures alone (mean MD and FA) predicted good outcome with 43% (25–60%) sensitivity and poor outcome with 60% (33–81%) sensitivity (Fig. 3). Prediction model three, combining the clinical model with mean MD and FA predicted good outcome with 93% (73–100%) and poor outcome with 85% (64–96%) sensitivity. The sensitivity analysis with model four, combining the clinical model with mean FA and %MD_450_ yielded similar results as model three, with prediction of good outcome with 97% (85–100%) sensitivity and poor outcome with 80% (57–95%) sensitivity (Fig. 3). Summarising, the addition of DTI parameters to the clinical model does not increase the sensitivity for prediction of good outcome. Sensitivity for the prediction of poor outcome without false positives increased. However, confidence intervals are overlapping.

## Discussion

Patients with poor neurological outcome after cardiac arrest show lower free water corrected MD, FA, and higher %MD_450_ than patients with good outcome. Exploratory prediction models suggest that these MRI parameters hold potential to add to multimodal outcome prediction of comatose patients after cardiac arrest.

Our results are aligned with previous studies, that have shown group differences and relevant predictive values of diffusivity measures. These studies showed utility for the apparent diffusion coefficient (ADC) derived from conventional DWI sequences [5, 6, 20, 36]. and for radial and axial diffusivity based on the DTI sequence [23, 37]. The MD measure derived in our study is equivalent to ADC, but while ADC is estimated from three diffusion directions, MD is more robustly estimated from 30 and 32 diffusion directions in our study. Aligned with the previous studies, we report a decrease in diffusivity in patients with poor outcome, most prevalent in the cortical grey matter. This can be explained by the high sensitivity to hypoxia of the GM. A post mortem study of patients who died two to five days after cardiac arrest (a timing similar to our MRI measurements) also showed a high prevalence of grey matter injury [38].

The additional predictive value of diffusion imaging appears to be high for the prediction of a poor neurological outcome and limited for the prediction of good outcome. Reliable prediction of poor outcome is important in clinical care, because it helps in determining if prolonged intensive life-supporting treatment is justified for a specific patient. When poor outcome cannot be predicted, treatment will generally be continued. Although (absence of) prediction of a good outcome will usually have a limited effect on the continuation of care, it supports communication between doctors and patients’ relatives and may aid in the decisions on the escalation of organ support [39].

Unlike previous studies, the MD measure in our study is free water corrected. Reported predictive values of ADC and MD vary greatly between studies. CSF contamination of the cortical grey matter is a possible cause for these discrepancies. CSF contamination and other free water influx are influenced by subject positioning, ventricle and subarachnoid space volume, hydration level, inflammation, and body temperature [40]. These factors can vary greatly across and within patients in the first days after cardiac arrest. This variation is reduced by using free water elimination techniques [40]. We found that free water-corrected MD yielded a higher effect size for discrimination between good and poor outcomes than uncorrected MD and allowed the identification of group differences in more areas of the brain.

While we found MD differences in the cortical grey matter, we did not find MD differences in the subcortical grey matter when comparing between patients with good and poor outcome. Differences in subcortical grey matter were observed in previous DWI studies [5, 6, 20], and were explained by the high metabolic demand of the deep grey nuclei [41]. However, a previous study on global ischaemia in a dog model found higher sensitivity to ischaemia in the cortex than in the basal ganglia [42]. This, together with the early and strict timing of our MRI scans possibly explains the lack of differences in the subcortical grey matter.

Differences in FA were most prominent in the white matter, with lower values in poor than in good outcome patients. Lower FA values in the white matter indicate less prominent directionality of diffusion along the white matter tract. Within the white matter, oligodendrocytes are responsible for myelinisation and are highly sensitive to hypoxia and hypoglycaemia [9, 43]. Swelling of the oligodendrocytes is an early response to ischaemia, followed by necrosis in more severe hypoxia [44]. This is followed by demyelination and swelling of the axons [44]. Both processes could explain the reduced FA in the white matter. The FA measure is less effective in identifying differences in the grey matter, since FA in healthy grey matter is already low. The effects of FA in the white matter complement those of MD in the grey matter, and both measures are thus useful to consider together in analyses.

Adding MD and FA measures to a prediction model based on parameters used in current clinical care improved predictive values in our cohort. Our predictive values are in line with predictive values previously reported by studies using ADC, MD, and FA [5, 6, 20, 22, 24, 36]. However, since the clinical parameters of the models have been used in decisions regarding WLST, we can only estimate the potential additional value of DTI to current clinical parameters. Absolute predictive values are prone to the self-fulfilling prophecy. In clinical practice, MRI will probably be performed when combined clinical and electrophysiological measurements and biomarkers preclude definite prediction of outcome.

Strengths of this study are the prospective study design, the early and strict timing of MRI and EEG measurements, and the multimodal approach. This study also has limitations. First, the sample size is relatively small, leading to a possible overestimation of our results, especially with regard to prediction. We also had to exclude four patients for the voxel-based analyses, due to pre-existent structural brain deficits, such as atrophy. Second, as in all studies on outcome prediction after cardiac arrest, we cannot fully exclude the influence of the self-fulfilling prophecy, since the withdrawal of life-sustaining treatment always results in poor outcome. However, MRI measures were not taken into account in decisions on treatment withdrawal. Third, the use of different MRI scanners could potentially affect our results, but this influence seemed to be small. Future studies could also improve the diffusion MRI acquisition to include multi-slice and multi-shell techniques which will make the acquisition faster and the model fit more robust, respectively.

Multiple steps are necessary before DTI imaging can be used as a prognosticator in daily clinical practice. First, the results of this study should be validated in a large, independent sample. Since each MRI scanner introduces a possible bias, vendor-specific cut-off values may improve results, but are difficult to validate. To prevent further bias, it is important to harmonise scan protocols and the processing of the data after acquisition [45]. Including the acquisition of a phase-encode reversed *b* = 0 image within the scan protocol could further improve data preprocessing, including a correction for field inhomogeneities and susceptibility artefacts. Validated and automated pipelines could then help clinicians to perform these analyses in a standardised fashion.

In conclusion, a combination of free water-corrected MD (sensitive to grey matter abnormalities) and FA (sensitive to white matter abnormalities) holds the potential to add to the prediction of good and poor outcome of comatose patients after cardiac arrest.

## Supplementary Information

ESM 1 (DOCX 4876 kb)

## Abbreviations

%MD_450_: Percentage of brain volume with MD < 450 × 10^–6^ mm^2^/s
CPC: Cerebral performance category
CSF: Cerebrospinal fluid
DTI: Diffusion tensor imaging
DWI: Diffusion-weighted imaging
FA: Fractional anisotropy
FSL: fMRI brain Software Library
MD: Mean diffusivity
OHCA: Out of hospital cardiac arrest
SSEP: Somatosensory evoked potential
WLST: Withdrawal of life-sustaining treatment

## Declarations

### Guarantor

The scientific guarantor of this publication is Prof. Dr. J. Hofmeijer.

### Statistics and biometry

No complex statistical methods were necessary for this paper.

### Methodology

• prospective

• diagnostic or prognostic

• multicentre study
