# Supplementary material for: Free water corrected diffusion tensor imaging discriminates between good and poor outcomes of comatose patients after cardiac arrest
Source: Eur Radiol. 2022 Nov 24;33(3):2139–48. doi: 10.1007/s00330-022-09245-w (PMC9935650; doi:10.1007/s00330-022-09245-w)
Supplement: Supplementary file 1 — (DOCX 4876 kb) [file 330_2022_9245_MOESM1_ESM.docx]

**Supplementary material**

*Free water corrected diffusion tensor imaging discriminates good and poor outcome of postanoxic coma*

*Keijzer et al.*

Table of contents

[Supplementary table 1. MRI sequence information 2](#_Toc100827870)

[Supplementary table 2. Overview of the EEG categories with corresponding EEG patterns for visual EEG classification. 3](#_Toc100827871)

[Supplementary table 3. Whole brain fractional anisotropy, mean diffusivity and percentage of brain volume with mean diffusivity below the threshold of 450*10^-6^ mm^2^/s, uncorrected for free water. 3](#_Toc100827872)

[Supplementary figure 1. Overview of screening and enrolment 4](#_Toc100827873)

[Supplementary figure 2 . Spatial distribution of differences in mean diffusivity before (A) and after (B) correction for free water 5](#_Toc100827874)

# **Supplementary table 1. MRI sequence information**

| **Parameter** | **Rijnstate hospital** | **Radboudumc** |
| --- | --- | --- |
|  |  |  |
| Scanner type | Philips Ingenia | Siemens Skyra |
| Field strength (Tesla) | 3.0 T | 3.0 T |
|  |  |  |
| **3D T1 sequence** |  |  |
| Imaging mode | 3D TFE | MPRAGE |
| Repetition time | “Shortest” (approximately 8.3 s) | 2.4 s |
| Echo time | “Shortest” (approximately 3.8 ms) | 3.4 ms |
| Inversion time | Not specified | 1.06 s |
| Flip angle | 8° | 8° |
| Voxel size (acquisition) | 1.0*1.0*1.0 mm | 0.9*0.9*1.0 mm |
|  |  |  |
|  |  |  |
| **DTI sequence** |  |  |
| Repetition time | 9.0 s | 9.7s |
| Echo time | 95 ms | 95 ms |
| Flip angle | 90° | 90° |
| Voxel size | 2*2*2mm | 2*2*2mm |
| b-values | b=0, b=1000 s/mm^2^ | b=0, b=1000 s/mm^2^ |
| Number of b=0 images | 1 | 1 |
| Number of diffusion directions | 32 | 30 |
|  |  |  |

# **Supplementary table 2. Overview of the EEG categories with corresponding EEG patterns for visual EEG classification.**

| **EEG category** | **Explanation** |
| --- | --- |
| **Suppressed patterns** | Continuously suppressed EEG, defined as an amplitude <*10µV* |
| **Synchronous activity on suppressed background patterns** | Synchronous burst-suppression**,** with generalized, abrupt-onset bursts or identical bursts with suppressed background activity (≥ 50% suppressions),  or generalized periodic discharges with suppressed background activity. |
| **Continuous patterns** | Continuous or nearly continuous activity: maximum amplitude ≥ 20µV, < 10% suppressions |
| **Other patterns** | Low-voltage (maximum amplitude 10-20 µV),  Epileptiform on other background,  Burst-suppression (heterogeneous with ≥ 50% suppressions),  Discontinuous (10-49% suppressions) |

# **Supplementary table 3. Whole brain fractional anisotropy, mean diffusivity and percentage of brain volume with mean diffusivity below the threshold of 450*10^-6^ mm^2^/s, uncorrected for free water.**

| **Parameter** | **Good outcome** | **Poor outcome** | **p-value** | **Effect size** |
| --- | --- | --- | --- | --- |
|  |  |  |  |  |
| Mean Diffusivity* | 807 [794-827] | 765 [709-836] | 0.06 | 0.84 (0.23-1.44) |
| Fractional Anisotropy | 0.22 ± 0.02 | 0.20 ± 0.02 | 0.03 | 0.66 (0.06-1.26) |
| Brain volume with MD<450* (%) | 0.6 [0.6-1.4] | 4.2 [0.9-9.5] | <0.01 | 0.95 ( 0.34-1.57) |

*Data are represented as n (%) for dichotomous variables, mean ± sd for normally distributed continuous variables and median [IQR] otherwise. Effect size is depicted as Cohen’s d (95% CI). MD: Mean Diffusivity;* *%MD_450_: brain volume with mean diffusivity below the threshold of 450*10^-6^ mm^2^/s; *MD x 10^-6^ mm^2^/s*


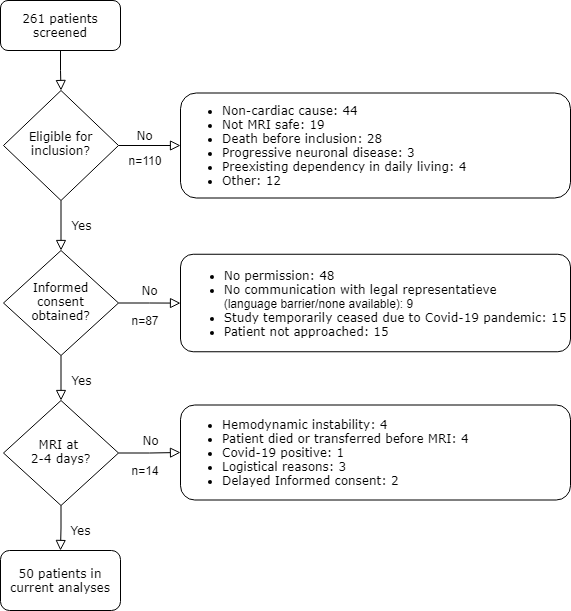


# Supplementary figure 1. Overview of screening and enrolment


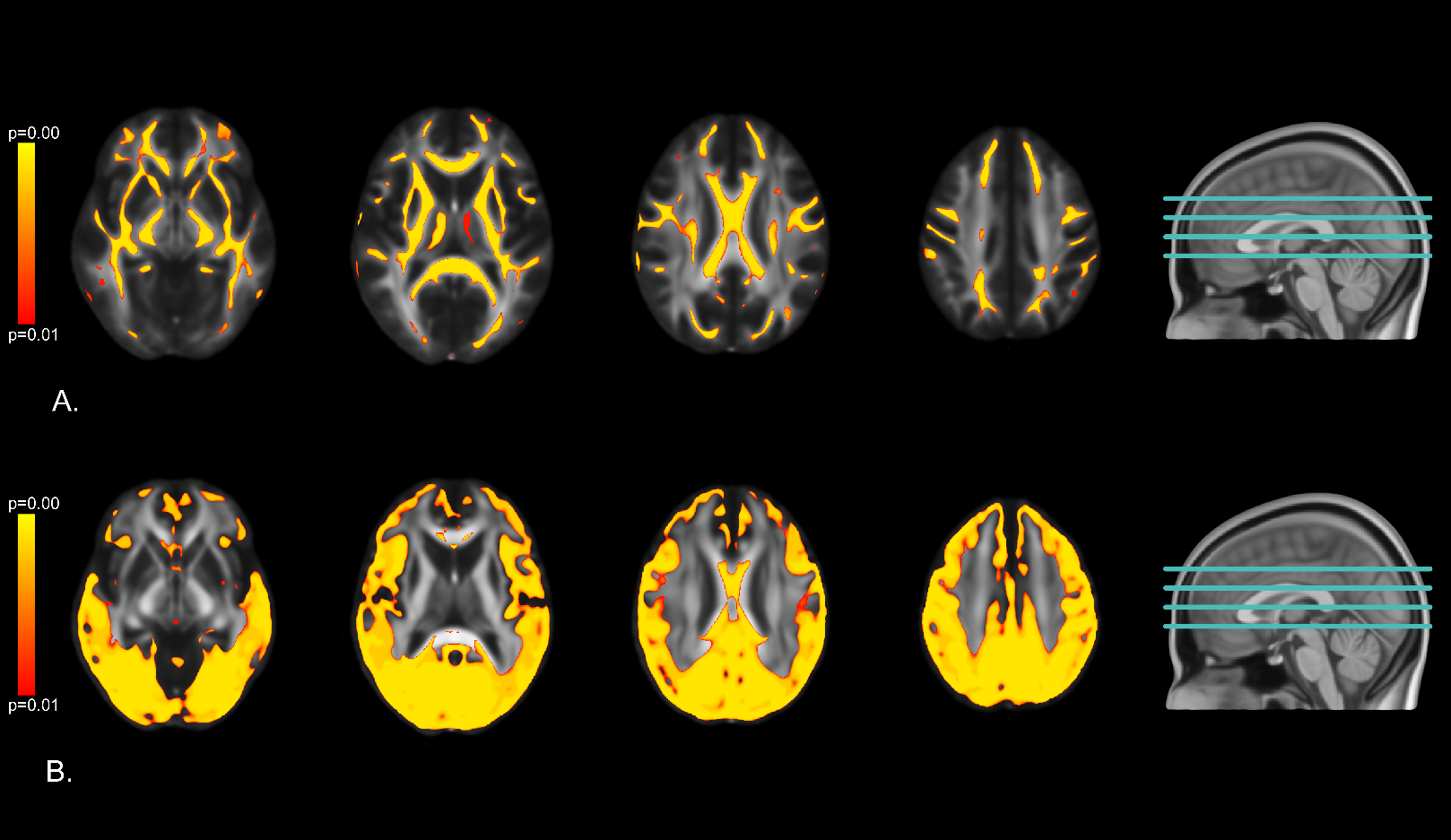

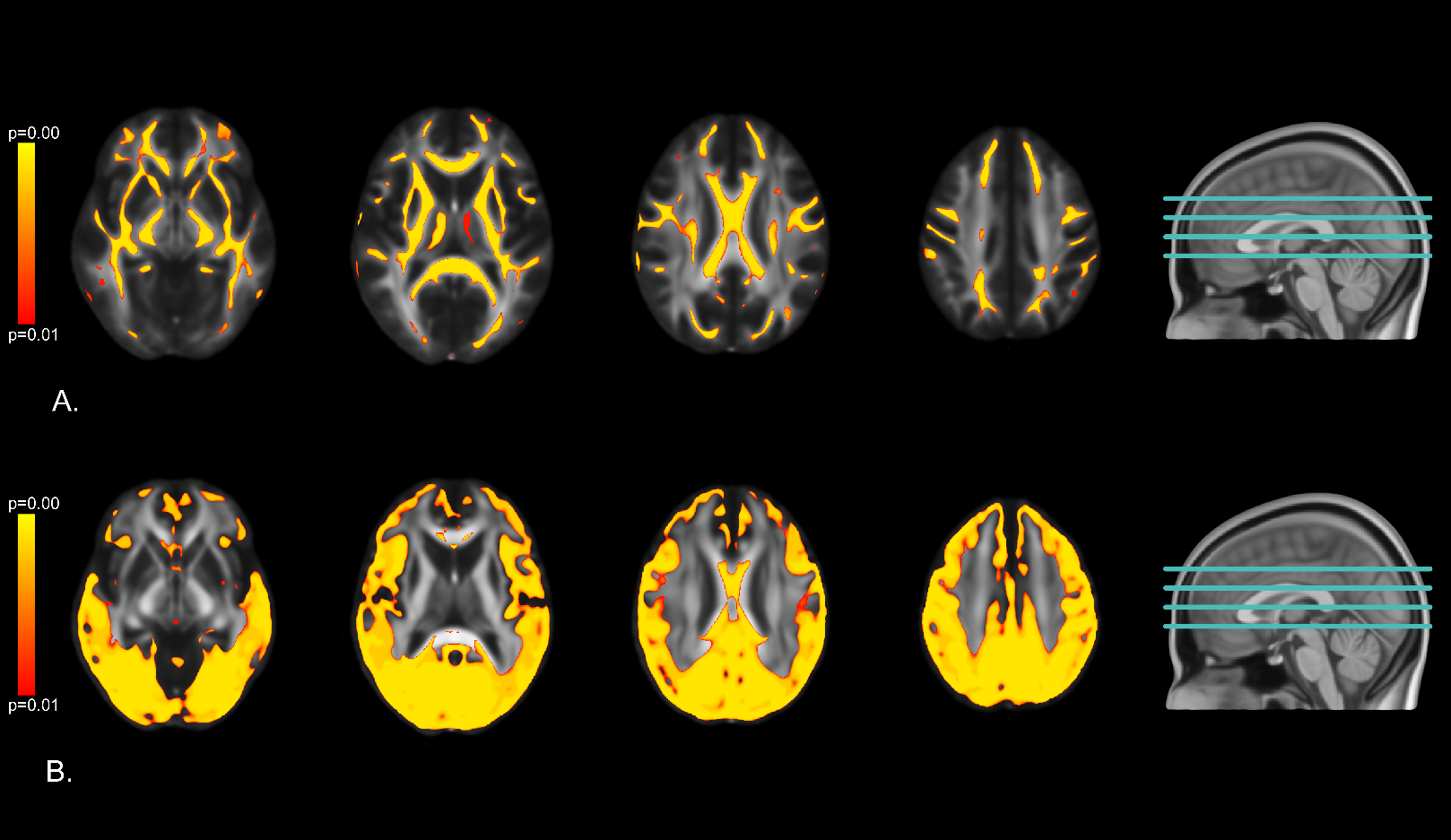


*Supplementary figure 2* *. Spatial distribution of differences in mean diffusivity before (A) and after (B) correction for free water. Coloured areas show brain areas where patients with poor neurological outcome show significantly lower values than patients with good neurological outcome (p<0.01).*
